# Ten Simple Rules for Writing a Reply Paper

**DOI:** 10.1371/journal.pcbi.1004536

**Published:** 2015-10-08

**Authors:** Mark P. Simmons

**Affiliations:** Department of Biology, Colorado State University, Fort Collins, Colorado, United States of America; Whitehead Institute, UNITED STATES

## Introduction

*Efforts to subject one’s own pet hypotheses to severe tests*, *to attempt to falsify them*, *are always warranted*. *The likelihood that one will impose unrealistically high standards is quite low*. *The same cannot be said for the standards that scientists set for the work of their opponents…*. *As unseemly as factionalism in science may be*, *it does serve a positive function*. *It enlists baser human motives for higher causes*.*—David L*. *Hull*, Science as a Process: An Evolutionary Account of Social and Conceptual Development of Science [1]

Have you read a paper or watched a presentation at a scientific conference with an utterly wrong or unsubstantiated conclusion? Have you participated in a discussion group wherein the group identified a fatal flaw in a recently published paper? The simple thing to do is discard the paper that you just read, ask a tough question at the end of the presentation, and make jokes about the paper with your colleagues at the end of the discussion, respectively. By the time students complete their PhD, they have probably seen instances of all three of these. But rather than stop there, it may be appropriate to invest substantial extra effort to write a formal reply.

## Rule 1: Determine If It Is Appropriate to Write a Reply

If your focus is on one or two papers, then a reply is the proper forum. However, if you want to address numerous related papers and advocate a shift towards better practices for an entire discipline, then you may be better off writing a perspectives paper for the applicable *Annual Reviews* or *Trends* journals (though these generally require invitations).

Writing replies is not a research program in and of itself, and you should not expect to make a successful career out of writing replies. You cannot get grants for writing replies, but you can make plenty of adversaries who go on to serve as your funding-agency program officers or panelists, editors, and reviewers. In the same way that you should carefully pick your fights, you should exercise discretion about when to write a reply.

If the original paper is scientifically uninteresting, then your reply will probably be equally uninteresting and not worth writing. One exception to this is when the original paper was published in a prominent journal. A classic example is Williamson’s [2] *Proceedings of the National Academy of Sciences of the USA* (PNAS) paper, wherein he asserted that larvae and adults did not evolve from a single common ancestor. This fantastic communicated submission from an NAS member was immediately rebutted by a direct submission from Hart and Grosberg [3]. That same year the editor in chief of PNAS [4] announced that the journal would stop accepting communicated submissions.

## Rule 2: Do Not Be Intimidated by Big Data—or Big Names

In this Age of Omics [5] (e.g., genomics, proteomics, and transcriptomics) and massive publicly available data repositories (e.g., Dryad, GenBank, and The Arabidopsis Information Resource), you should not assume that the original authors’ biologically implausible yet impressively well-supported conclusions are correct. Big data have the potential to create impressive *p*-values for incorrect conclusions even when there is a small but consistent underlying bias. Find the underlying bias, and the impressive *p*-values may vanish. In this context, understanding the limitations of the analytical methods applied by the original authors and knowing what patterns to look for while examining their data are more important than being able to feed the data through bioinformatic pipelines. Too many authors rely upon summary statistics generated by pipelines.

Early in this Age of Omics, the International Human Genome Sequencing Consortium [6] claimed to have identified 223 genes that had been laterally transferred from bacteria to vertebrate nuclear genomes based on Protein BLAST scores. By contrast, Salzberg et al. [7] asserted that the earlier claim could be more plausibly explained by rate heterogeneity among lineages together with taxonomic undersampling and gene loss in nonvertebrate eukaryotic genomes. Salzberg et al.’s [7] successful rebuttal was based on their understanding the limits of BLAST searches, making appropriate null hypotheses, and examining the original data in a phylogenetic context; they relied on first principles rather than attempting to develop new algorithms or sequence new genomes.

In the same manner that you should not be intimidated by big data, you should not be intimidated by big names either. This is not a new idea—it’s widespread on buttons and bumper stickers: “Question Authority.” Wenzel [8] provided the following fun example from his undergraduate studies that I tell all of my students:

For example, when I was studying thermodynamics in introductory physics in 1977, my professor (a Nobel Laureate) explained how the rate of heat production and heat loss limits mammals to be no smaller than a shrew, and of course, shrews are the smallest mammals. A student in the front row asked “Dr. Purcell, what about baby shrews?” The professor’s jaw dropped; he had never thought of that.

## Rule 3: Stick to the Facts

Do not get personal by writing inflammatory statements. Instead, let the evidence speak for itself—you are writing for an intelligent, specialist audience that is capable of reaching its own conclusions. Furthermore, do not write in a righteous manner, because there is always the possibility that you are wrong. Your reply will become part of the permanent scientific record, and a cute but false remark might still be talked about by your colleagues 20 years hence.

You may have fond memories of reading flamboyant reply papers (e.g., [9,10]). But unless you are at the top of your field and have a close relationship with the editor, do not expect to get away with it because you will probably be forced to remove the flamboyant text by the reviewers and editors. Even if you are at the top of your field, you still have to live with the long-term consequences of upsetting the original authors. For example, as of 2015, Sudhir Kumar is the editor in chief of *Molecular Biology and Evolution*, while William Martin is the editor in chief of its sister journal, *Genome Biology and Evolution*. These two editors are expected to coordinate these journals, irrespective of their aggressive replies to each other [9,11].

## Rule 4: Expect a Reply to Your Reply

Your reply will be held to a very high standard by the original authors, and rightfully so. Expect to be quoted, with “[sic]” included, where applicable, so carefully proofread your manuscript. Scientific papers should always be readable literally. However, that fact is especially relevant to how the original authors may reply to you. If you overstate your case by making absolute assertions, then expect their reply to focus on that. You are the one picking a fight, so you better win it.

## Rule 5: Make Points of General Interest

Try to make points of general interest rather than just writing for the small target audience who read the original paper. This approach is relevant for the sake of getting your reply accepted into a prominent journal (ideally the same journal in which the original paper was published) and having your reply cited. If all you do in your reply is successfully refute the original paper, then you have made an important scientific contribution, but few people will ever cite your reply (or the original paper) because the case is closed.

Having your reply published in the same journal as the original paper helps you reach the readers of the original paper. If these readers do not read your reply, then they may think that the applicable conclusions from the original paper are still valid. In addition to your choice of journal, another way to increase the prominence of your reply is to make it a community effort by inviting colleagues to join your reply as coauthors. For example, Melissa Luckow [12] demonstrated wide support for her viewpoint that the type species of the legume genus *Acacia* should not be moved from Africa to Australia by teaming up with 36 other botanists whose work would be affected by the taxonomic change proposed by Orchard and Maslin [13].

If you develop a reputation for writing informative reply papers, then many of your colleagues will read your reply even if they have not read the original paper so that they can better understand the issues being discussed. Help your readers out by objectively summarizing the original paper, providing the necessary context, and, if applicable, clarifying the assertion(s) made by the original authors in your introduction.

## Rule 6: Be Positive Too

Rather than just pointing out flaws, present an alternative analytical method or empirical conclusion that improves upon the method or conclusion from the original paper. As Theodore Roosevelt famously stated in his 1910 speech at the Sorbonne [14], “It is not the critic who counts; not the man who points out how the strong man stumbles, or where the doer of deeds could have done them better. The credit belongs to the man who is actually in the arena….”

There are many classic replies for which the original papers have been largely forgotten but the reply lives on because the authors of the reply presented an important novel alternative. Nixon [15] demonstrated the efficiency of his novel Parsimony Ratchet by showing that it found shorter phylogenetic trees with dramatically less computing power (a desktop in less than two hours versus Sun workstations in months) than Rice et al.’s [16] traditional tree search on a 500-taxon dataset. Nixon [15] rendered a direct reply to Rice et al. [16] unnecessary because he showed that their tree-search strategy was obsolete. According to Web of Science, Nixon [15] was cited 86 times in 2014, whereas Rice et al. [16] was not cited at all.

## Rule 7: Only Present Your Best Arguments

Do not present all possible arguments. If you do so, then a reply by the original authors might only address your weakest arguments, yet casual readers will think that your reply has been invalidated because the majority of your arguments have been successfully rebutted or at least muddled. Instead focus on your most important arguments and clearly itemize them so they cannot be overlooked by the original authors should they choose to write their own reply. You do not have to refute every point made in the original paper to write an effective reply. It is enough to refute their primary conclusion(s) rather than pedantically going after every point.

## Rule 8: Use the Authors’ Own Arguments against Them

If applicable, demonstrate that the original authors’ conclusion is falsified by their own criteria rather than just evaluating their conclusion using your own criteria. James S. Farris has published numerous such replies. In perhaps his most important reply, Farris [17] demonstrated that most parsimonious trees, rather than trees constructed using overall similarity, have the highest information content. By doing so, Farris demonstrated that the primary goal of phenetics [18] was best solved using cladistic methods, thereby effectively undermining the justification for using phenetics for taxonomic classifications.

## Rule 9: Present the Logical End Point of Faulty Arguments

If applicable, demonstrate that the original authors’ argument is untenable when it is taken to its logical conclusion. For example, Mollison [19] demonstrated that the Lotka-Volterra equations, when applied to predators and their prey [20], can require that the prey population recover from a tiny fraction (10^−18^) of an individual, which Mollison famously referred to as an “atto-fox” (atto being the prefix for 10^−18^ and fox being the [rabies virus’] prey in his empirical example).

## Rule 10: Demonstrate That Conclusions Can Be Explained by the Null Hypothesis

If the original authors’ assertions are untestable, tautological, or can be equally well explained by the null hypothesis, then they are uninformative. Connor and Simberloff [21] demonstrated that one or more of these flaws apply to all seven of Diamond’s [22] ecological assembly rules, thereby refuting the bases for Diamond’s inductive conclusion that interspecific competitive exclusion is a primary factor in determining bird communities.
